# Probiotic potential and safety assessment of bacteriocinogenic *Enterococcus faecalis* CAUM157

**DOI:** 10.3389/fmicb.2025.1563444

**Published:** 2025-07-01

**Authors:** Arxel G. Elnar, Geun-Bae Kim

**Affiliations:** Department of Animal Science and Technology, Chung-Ang University, Anseong, Republic of Korea

**Keywords:** *Enterococcus faecalis*, genome analysis, probiotic properties, bacteriocin, antimicrobial activity, multi-locus sequence typing, niche factors

## Abstract

**Introduction:**

Over the past few years, the genus *Enterococcus* has been implicated as both probiotic and pathogenic bacteria, making it challenging to introduce enterococcal probiotic candidates. Based on rigid case-by-case analysis, some enterococcal strains have been developed as probiotics, exhibiting various beneficial activities that promote the health of the host. In this study, the probiotic potential of *Enterococcus faecalis* CAUM157 (KACC81148BP), isolated from raw cow’s milk, was examined, leveraging its bacteriocin production for potential control of pathogenic and spoilage-associated bacteria.

**Methods:**

The probiotic properties of *Enterococcus faecalis* CAUM157 were evaluated using a combination of genomic analyses and *in vitro* assays. In addition, various in silico analyses were performed to assess the safety of the strain and correlate its genotype with the observed phenotypic characteristics.

**Results and discussion:**

Genomic analyses revealed virulence factors associated with adhesion, biofilm formation, and anti-phagocytosis. Various enzymes and antimicrobial resistance genes that confer resistance to aminoglycosides, lincosamides, macrolides, streptogramins A and B, and tetracyclines were also identified. Although generally regarded as detrimental, virulence factors are crucial to colonization, niche establishment, and subsequent manifestation of the beneficial effects of the strain, as evident in other probiotic lactic acid bacteria. Notably, CAUM157 was sensitive to clinically important antibiotics like ampicillin (MIC, 4.0 µg/mL) and vancomycin (MIC, 1.0 µg/mL), congruent with its ST21 MLST typing. CAUM157 survived in acidic conditions (pH 3.0 and pH 2.0) with 100.72 ± 0.20% and 97.28 ± 2.19% survival rates, respectively, and showed high survival rates when exposed to 0.3% (104.16 ± 3.42%) and 0.5% (90.65 ± 1.22%) bile extract, attributed to the enzymatic activity of bile salt hydrolase. CAUM157 also exhibited robust auto-aggregation and co-aggregation when interacting with *Listeria monocytogenes*. Finally, the ability to produce a broad-spectrum bacteriocin in conjunction with other factors indicates a potentially efficient mechanism for mitigating the pathogenicity of detrimental bacteria, including *Staphylococcus aureus*, *Clostridium perfringens*, and *Streptococcus mutans*.

**Conclusion:**

*Enterococcus faecalis* CAUM157 survived exposure to artificial gastric conditions and exhibited robust auto- and co-aggregation capacity with *Listeria*. Additionally, the ST21 MLST typing of the strain, along with the identified niche factors and the lack of resistance to vancomycin and ampicillin, highlights its apparent safety. The results of this study suggest that strain CAUM157 may be a potential probiotic candidate in the food and feed industries.

## 1 Introduction

*Enterococcus* species are commonly associated with numerous health benefits in humans and animals. As members of the lactic acid bacteria (LAB) group, they can stimulate the immune response, improve gut barrier function, and limit the growth of potentially harmful bacteria (i.e., foodborne pathogens and periodontitis-causing pathogens) by producing antimicrobial substances such as bacteriocins (Hanchi et al., 2018; Baccouri et al., 2019; Park et al., 2021). They are facultative-anaerobic and highly adaptable species that can survive and colonize the gastrointestinal tract of their host due to their resistance to low pH and tolerance to bile salts, allowing them to exhibit probiotic or beneficial health effects (Begley et al., 2006; Krawczyk et al., 2021). Furthermore, enterococci enhance the breakdown of non-digestible feed components such as complex polysaccharides, leading to improved digestion and nutrient utilization, thereby improving the overall health and performance of the animal host (Hanczakowska et al., 2016; Hu et al., 2019).

Numerous studies have documented the advantages of administering *Enterococcus* spp. as a supplement to livestock such as cattle, poultry, swine, and even aquatic organisms such as fish (Braïek and Smaoui, 2019; Shehata et al., 2020). The beneficial health effects include better growth performance, enhanced feed efficiency, maintained intestinal villi morphology, enhanced immunity, optimal gut microbiota composition, and overall promotion of eubiosis (Allameh et al., 2017; Wang et al., 2019; Maake et al., 2021). However, this genus remains controversial because of the widespread occurrence of horizontal gene transfer (HGT), which imparts virulence and resistance to several antimicrobial agents across distantly related species (Zaghloul and El Halfawy, 2022; Im et al., 2023).

Nevertheless, by conferring a competitive advantage over pathogens susceptible to the same antimicrobials, the intrinsic resistance of enterococci, including those commonly used in livestock and poultry (e.g., penicillin, tetracycline, and ciprofloxacin), may potentially be beneficial in probiotic applications (Hofacre et al., 2013; Gilmore et al., 2020). Certain probiotic strains have been carefully evaluated for antibiotic resistance to be used in conjunction with antibiotics treating specific conditions (Hammad and Shimamoto, 2010). In this regard, it is vital to emphasize that the existence of antimicrobial resistance genes (ARGs) constitutes a reservoir of resistance for potential gut pathogens and raises serious safety concerns (Gueimonde et al., 2013). Therefore, a thorough, strain-level evaluation of probiotic safety before commercial development and manufacturing is critical. Once the strain enters the market, strict monitoring is required to ensure product quality and safety (Imperial and Ibana, 2016). Furthermore, the presence of acquired ARGs requires the identification of the surrounding genes (in the genomic context) and other factors that can influence their transferability.

Recent advances in molecular epidemiology facilitated the distinction of nosocomial and commensal enterococcal strains based on their genotypes (Werner et al., 2011; Neumann et al., 2019; Zhong et al., 2019). This calls into question the current guidelines and suggestions for differentiating potentially probiotic and pathogenic *Enterococcus* strains. Among various strategies, whole genome analyses have been used to identify ARGs and virulence factors, a crucial step in evaluating the strain’s safety (Heo et al., 2018; Qureshi et al., 2020; Pell et al., 2021). Moreover, various *in silico* analyses, which are particularly useful for characterizing probiotic candidates, can predict the pathogenicity and overall safety of a strain based on its genomic properties (Neumann et al., 2019). Prior to these scientific developments, strains of enterococci have already been used as food additives and supplements based on careful case-by-case assessment, as permitted by organizations such as the European Food Safety Authority (EFSA), the Advisory Committee on Novel Foods and Processes (ACNFP) in the United Kingdom, and the Food Standards Agency (FSA) (Rychen et al., 2018; Graham et al., 2020; Ferchichi et al., 2021). Some enterococci used in the therapeutic treatment of irritable bowel syndrome, recurrent chronic sinusitis, or bronchitis include Symbioflor1 (SymbioPharm, Germany), Cylactin® (Hoffman-La Roche, Switzerland), Fargo 688® (Quest International, Netherlands), and ECOFLOR (Walthers Health Care, Netherlands) (Domann et al., 2007). The rise in the number of developed probiotic enterococci may facilitate changes in the Qualified Presumption of Safety (QPS) or Generally Recognized as Safe (GRAS) status of *Enterococcus* (Suvorov, 2020; Dapkevicius et al., 2021).

In this study, we evaluated the genotypic and phenotypic properties of the facultatively anaerobic *Enterococcus faecalis* CAUM157, a bacteriocin-producing strain isolated from raw cow’s milk (Elnar et al., 2020), for potential development as a probiotic candidate, focusing on pathogen control. The genome of CAUM157 was screened for antimicrobial resistance genes and other potentially harmful factors, including virulence factors, genomic islands, and integrated plasmids. Further, the probiotic characteristics of the strain were examined *in vitro*, focusing on its carbohydrate fermentation profile, resistance and tolerance to gastric conditions, and ability to co-aggregate with *Listeria monocytogenes*. Finally, the inhibitory activity of its bacteriocins was evaluated against Gram-negative and Gram-positive bacterial species.

## 2 Materials and methods

### 2.1 Comparative genomic analyses

The whole genome of *Enterococcus faecalis* CAUM157, a strain isolated from raw cow’s milk, was sequenced, characterized, and deposited into NCBI database with the accession number GCA_014805465.1 (Elnar et al., 2020). The genome was examined and compared with that of other previously published *E. faecalis* strains: V583 (GCA_000007785.1), UK045 (GCA_021610105.1), ATCC 29212 (GCA_001999625.1), T5 (GCA_000393015.1), D32 (GCA_000281195.1), OG1RF (GCA_000172575.2), and Symbioflor1 (GCA_014353145.1). Whole genome comparison was performed using the BLAST Ring Image Generator (BRIG) 0.95 (Alikhan et al., 2011) with the genome of *E. faecalis* V583 as a reference. Virulence factors and antimicrobial resistance genes (ARGs) were annotated based on GenBank files to generate a circular map of the genome.

The Virulence Factors of Pathogenic Bacteria Database (VFDB) was used to confirm the presence of virulence factors through a BLAST search (Chen et al., 2005). The overall pathogenicity was predicted using PathogenFinder 1.1 (Cosentino et al., 2013), and the presence of ARGs was determined using ResFinder 4.0 (Florensa et al., 2022), and Resistance Gene Identifier 5.2.0 (RGI) using the Comprehensive Antibiotic Resistance Database (CARD) (Alcock et al., 2023). The presence of genomic islands was determined using IslandViewer4 (Bertelli et al., 2017). Integrated plasmids were detected using PlasdmidFinder 2.0 (Carattoli et al., 2014) and the features present in the plasmid obtained from the NCBI database were searched in the CAUM157 genome using the EzBio BLAST program (Chalita et al., 2024). The Multi-Locus Sequence Typing (MLST) profile was predicted using the MLST 2.0 server (Larsen et al., 2012).

### 2.2 Antimicrobial susceptibility testing (AST)

The antimicrobial susceptibility profile of *E. faecalis* CAUM157 was determined using the broth microdilution method (CLSI, 2020). The antibiotics listed in Table 1 were prepared at an initial concentration of 2,048 μg/mL in Cation-adjusted Mueller-Hinton Broth (CAMHB, Merck, Germany). Sterile 96-well plates were filled with 100 μL of sterile CAMHB in each well. Then, 100 μL of each antibiotic (2,048 μg/mL) was added to column 1 and serial two-fold dilution was performed until column 10 (final concentration, 2 μg/mL). Then, CAUM157 strain cultured overnight in De Mann, Rogosa, Sharpe broth (MRS, BD Difco) + 0.05% L-cysteine-HCl (Sigma Aldrich) (cys-MRS) was harvested by centrifugation (8,000 × g, 4°C, 10 min) and washed twice in 1 × phosphate buffered saline (PBS) before inoculating 100 μL in CAMHB to a final cell concentration equivalent to 0.5 McFarland standard (Baker et al., 1983). Column 11 was inoculated with CAUM157 without antibiotics and column 12 was filled with uninoculated CAMHB, serving as negative and blank control, respectively. The plates were incubated statically at 37°C and cell growth was determined by measuring the absorbance at 600 nm using an INNO spectrophotometer (LTEK Co., Ltd.). Susceptibility or resistance was interpreted based on the minimum inhibitory concentration (MIC) cutoff for resistance set by the Clinical and Laboratory Standards Institute (CLSI) for *Enterococcus* spp. (CLSI, 2020).

**Table 1:** Antimicrobial resistance profile of CAUM157 based on broth microdilution assay.

Antimicrobial	CLSI Cutoff (μg/mL)	MIC (μg/mL)	Phenotype
Ampicillin	≥ 16	1	Susceptible
Vancomycin	≥ 32	4	Susceptible
Ciprofloxacin	≥ 4	≥ 512	Resistant
Erythromycin	≥ 8	≥ 512	Resistant
Tetracycline	≥ 16	≥ 512	Resistant
Clindamycin*	-	≥ 512	Resistant
Kanamycin*	-	≥ 512	Resistant
Streptomycin*	-	≥ 512	Resistant

*Intrinsic resistance.

### 2.3 Carbohydrate fermentation profile

The carbohydrate fermentation profile of *E. faecalis* CAUM157 was determined using the API 50CH kit (BioMérieux, Cambridge, USA) following the manufacturer’s instructions. First, a cell suspension of CAUM157 was prepared in 0.85% NaCl, equivalent to the 2.0 McFarland standard, and inoculated in API CHB medium to achieve cell concentration equivalent to 0.5 McFarland standard (Baker et al., 1983). The cupules were then filled with cell suspension, and the strips were incubated accordingly. A color change from purple to yellow (or purple to black for esculin) indicated carbohydrate fermentation. The fermentation profile of CAUM157 was compared that reported previously for other *E. faecalis* and *E. faecium* strains.

### 2.4 Bile salt hydrolase activity

The activity of bile salt hydrolase (BSH) enzyme was confirmed using washed cells prepared from overnight cultures of CAUM157. Briefly, washed cells were prepared at 5 × and 1 × cell concentrations in 1 × PBS (approx. 5 × 10^9^ and 1 × 10^9^ CFU/mL, respectively). Then, 10 μL of cell suspension was inoculated in 190 μL of BSH reaction buffer (50 mM, pH 5.5 sodium-phosphate buffer, 0.1% CaCl_2_, 10 mM dithiothreitol (DTT), and 10 mM Na-GDCA). The tubes were incubated in a 37°C water bath until a white precipitate was formed, which indicated bile salt deconjugation. The time required for the formation of white precipitates was recorded.

### 2.5 Acid and bile salt tolerance

The survival of CAUM157 cells under gastric conditions was also assessed. To determine the ability of the strain to survive under acidic conditions, CAUM157 cells were exposed to artificial gastric juice (AGJ) comprising 0.2% pepsin (Roche Diagnostics, USA) and 0.35% NaCl (Daejung Chemicals, Korea) set at pH 2.0 and 3.0 by adding 3 N HCl. Briefly, a 5 mL overnight culture of CAUM157 was harvested by centrifugation (8,000 × g, 4°C, 10 min), washed twice with 1 × PBS, and resuspended in an equal volume of PBS to achieve a 1 × cell concentration. Then, 1% (v/v) of washed cells was inoculated in 5 mL AGJ (pH 2.0 or 3.0) and incubated at 37°C. Viable cell counts were determined by spread plating on MRS agar after 0, 15, 30, 60, and 120 min of exposure and were reported as log(CFU mL^−1^).

Survival in the presence of bile was determined by inoculating 1% washed cells (v/v) in 5 mL cys-MRS supplemented with either 0.5% or 0.3% porcine bile extract (PBE, Sigma Aldrich). The cultures were incubated at 37°C and viable cell counts were determined every 6 h for 24 h. Survival rates at each timepoint were calculated using the following formula:


$${\text{Survival Rate}}_{t}\;(\%)=\frac{\log(\mathrm{CFU}\;mL_{t}^{-1})}{\log(\mathrm{CFU}\;mL_{t0}^{-1})}\times 100$$

in which CFU mL^−1^_t_ is the cell concentration after exposure at each timepoint and CFU mL^−1^_t0_ is the cell concentration of the initial inoculum. Experiments were performed thrice (in triplicate).

### 2.6 Auto-aggregation and co-aggregation

Auto- and co-aggregation of *E. faecalis* CAUM157 were determined following a previously established protocol (Todorov et al., 2011). First, CAUM157, and *Listeria monocytogenes* ATCC 19111, ATCC 19114, and ATCC 19115, were cultured in cys-MRS and TSB, respectively, at 37°C for 24 h. Cells were harvested, washed with sterile distilled water, and resuspended in sterile saline water (0.85% NaCl) to a final cell concentration of OD_660nm_ = 0.3, using an INNO microplate spectrophotometer.

The degree of auto-aggregation was determined by transferring 1 mL of cell suspension in a 1.75 mL microfuge tube and incubating at ambient room temperature (25°C) for 60 min, followed by centrifugation at 720 × g for 2 min. The supernatant was transferred to a 96-well plate, each well containing 250 μL, and the absorbance at 660 nm was measured. Auto-aggregation was calculated using the following formula:


$$\text{Auto}-\text{aggregation}\;(\%)=\frac{OD_{0}-OD_{60}}{OD_{0}}\times 100$$

where OD_0_ is the absorbance at 600 nm before incubation and OD_60_ is the absorbance of the supernatant at 600 nm after 60 min of incubation.

The degree of co-aggregation was determined by combining 500 μL cell suspensions of CAUM157 and *L. monocytogenes* strains in a 1.75 microfuge tube and incubating at ambient room temperature (25°C) for 60 min, followed by centrifugation at 720 × g for 2 min. The supernatant was transferred to a 96-well plate, each well containing 250 μL, and the absorbance at 660 nm was measured. Co-aggregation was calculated using the following formula:


$$\mathrm{Co}-\text{aggregation}\;(\%)=\frac{OD_{\mathrm{Tot}}-{\mathrm{OD}}_{S}}{OD_{\mathrm{Tot}}}\times 100$$

in which OD_Tot_ is the absorbance at 600 nm immediately after the strains were mixed and OD_S_ is the absorbance of the supernatant at 600 nm after 60 min of incubation. The experiments were performed thrice, in triplicate.

### 2.7 Antimicrobial activity against pathogens

The antimicrobial activity of CAUM157 against Gram-positive and Gram-negative bacteria was investigated using a spot-on-lawn assay, as previously described (Castilho et al., 2019). Briefly, a lawn of the test strain was prepared by spread-plating 100 μL of 24 h culture on respective agar media. Then, 10 μL of purified bacteriocins at decreasing concentrations (30, 20, 10, 8, 4, 2, 1 μg/mL) was spotted. The plates were incubated and the presence of an inhibition zone was observed. The lowest concentration of bacteriocin that showed complete inhibition was considered the MIC for the test organism.

### 2.8 Statistical analyses

Data are expressed as the mean ± standard error calculated over three independent experiments performed in triplicate and analyzed using one-way ANOVA with GraphPad Prism ver. 9.5.1.

## 3 Results

### 3.1 Comparative genomic analysis

**Figure 1:**
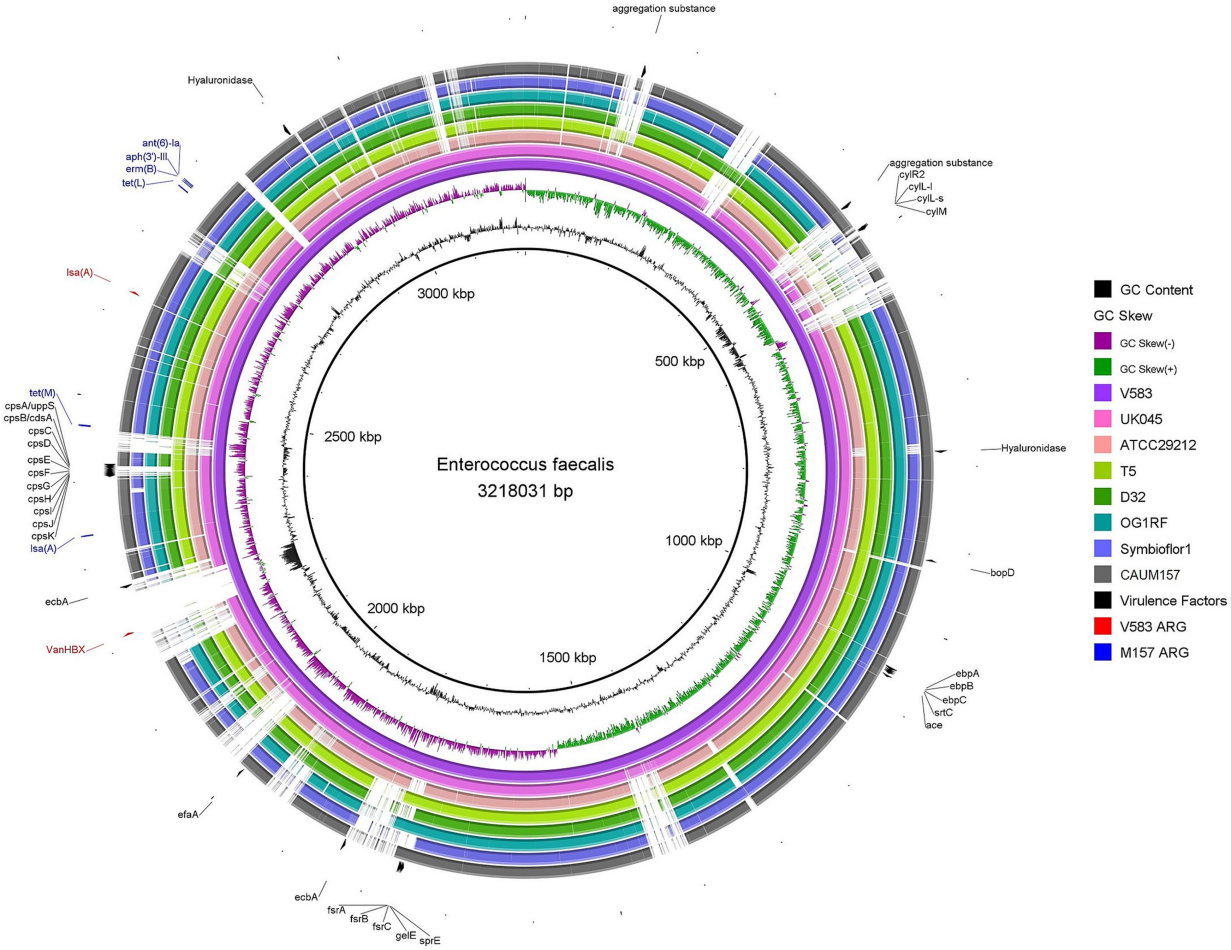
Circular comparison of *Enterococcus faecalis* genomes using BRIG. The rings, from innermost outward, represent GC content (black line), GC skew (green and purple lines), genome of strains V583 (light purple), UK045 (pink), ATCC 29212 (peach), T5 (light green), D32 (green), OG1RF (teal blue), Symbioflor1 (lavender), and CAUM157 (gray). Feature annotations represent virulence factors (black) and antimicrobial resistance genes of V583 (red) and CAUM157 (blue).

Comparative genomic analysis was performed to investigate the similarities and differences between the genome of *Enterococcus faecalis* CAUM157 and other published strains, namely V583, UK045, ATCC 29212, T5, D32, OG1RF, and Symbioflor1. Multiple whole genome comparisons were performed by generating circular maps of the genomes obtained from the NCBI database. The virulence factors and ARGs were annotated based on the reference strain (V583), as shown in Figure 1. Notably, most strains shared the same virulence factors and ARGs despite differences in their origin and virulence. Except for strain V583, all tested strains lacked vancomycin resistance genes. The observed genetic profiles suggest that these virulence factors and ARGs are conserved across species and play a critical role in survival and contribute to niche establishment of the species.

### 3.2 Virulence factors and antimicrobial resistance genes

The genome of *E. faecalis* CAUM157 was screened for virulence factors, ARGs, and transposable elements. *In silico* genetic analysis revealed the presence of antimicrobial resistance genes predicted to confer resistance to aminoglycosides, lincosamides, macrolides, streptogramins, and tetracycline (Table 2). Notably, the strain CAUM157 does not harbor genes for vancomycin resistance, which is of prime concern in clinical settings because of the strong association between vancomycin-resistant *Enterococcus* (VRE) strains and increased morbidity and mortality. Furthermore, 18 genes related to virulence factors were identified (Table 3). Strains CAUM157, D32, OG1RF, and V583 share common virulence factors associated with adherence (*ace, ebpA, ebpB, epbC, srtC,* and *efaA*), anti-phagocytosis (*cpsA/uppS* and *cpsB/cdsA*), biofilm formation (*bopD, fsrA, fsrB,* and *fsrC*), and several enzymes, including gelatinase (*gelE*), hyaluronidase (*hylB*), and serine protease (*sprE*). Only strain V583 harbored genes encoding the cytolysin toxin, which is considered one of the most widely distributed peptide toxins. Multilocus sequence typing analysis (MLST) revealed the presence of housekeeping genes *aroE* (shikimate dehydrogenase), *gdH* (glutamate dehydrogenase), *gki* (glucokinase), *gyd* (glyceraldehyde-3-phosphate dehydrogenase), *pstS* (phosphate-binding protein), *xpt* (xanthine phosphoribosyltransferase), and *yqiL* (putative acetyltransferase) genes (Table 4). Based on this, strain CAUM157 was classified as sequence type 21 (ST21).

**Table 2:** Predicted antimicrobial resistance genes in CAUM157 genome.

Gene	Predicted antimicrobial resistance phenotype	Class
*aph*(3′)-III^a^	Amikacin	Aminoglycoside
	Butisosin	
	Isepamicin	
	Kanamycin	
	Lividomycin	
	Neomycin	
	Paromomycin	
	Ribostamycin	
*ant*(6)-Ia^a^	Streptomycin	
*lsa*(A)^b^	Clindamycin	Lincosamide
	Lincomycin	
*erm*(B)^a^	**Erythromycin**	Macrolide
*lsa*(A)^b^	Dalfopristin	Streptogramin A
	Pristinamycin IIA	
	Virginiamycin M	
*erm*(B)^a^	Pristinamycin IA	Streptogramin B
	Quinupristin	
	Virginiamycin S	
*tet*(L)^a^	Doxycyline	Tetracycline
	**Tetracycline**	
*tet*(M)^a^	Minocycline	

a Percent identity (%ID) is 100; perfect match.

b Query length is shorter than resistance gene length; non-perfect match.

ARGs were identified using ResFinder 4.0 and Resistance Gene Identifier 5.2.0 (RGI) using the Comprehensive Antibiotic Resistance Database (CARD).

The minimum percentage of identity between the best matching resistance gene in the database and query was set at 90%. The minimum length of coverage overlaps for hit detection was set at 60%.

Antimicrobials in bold are specific for *Enterococcus faecalis*.

**Table 3:** Virulence factors among different strains of *Enterococcus faecalis* and *E. faecium*.

VFclass	Virulence factors	Related genes	*E. faecalis* CAUM157	*E. faecalis* D32	*E. faecalis* OG1RF	*E. faecalis* V583	*E. faecium* DO
Adherence	AS	Undetermined				+	
		Undetermined				+	
		asa1				+	
		prgB/asc10	+			+	
	Ace	*Ace*	+	+	+	+	
	Acm	*Acm*					+
	Ebp pili	*ebpA*	+	+	+	+	+
		*ebpB*	+	+	+	+	+
		*ebpC*	+	+	+	+	+
		*srtC*	+	+	+	+	+
	EcbA	*ecbA*				+	+
	EfaA	*efaA*	+	+	+	+	+
	Esp	*Esp*	+				
	Scm	*Scm*					+
	SgrA	*sgrA*					+
Antiphagocytosis	Capsule	*cpsA/uppS*	+	+	+	+	+
		*cpsB/cdsA*	+	+	+	+	+
		*cpsC*				+	
		*cpsD*				+	
		*cpsE*				+	
		*cpsF*				+	
		*cpsG*				+	
		*cpsH*				+	
		*cpsI*				+	
		*cpsJ*				+	
		*cpsK*				+	
Biofilm formation	BopD	*bopD*	+	+	+	+	+
	Fsr locus	*fsrA*	+	+	+	+	
		*fsrB*	+	+	+	+	
		*fsrC*	+	+	+	+	
Enzyme	Gelatinase	*gelE*	+	+	+	+	
	Hyaluronidase	Undetermined	+	+	+	+	
		Undetermined	+	+	+	+	
	SprE	*sprE*	+	+	+	+	
Toxin	Cytolysin	*cylA*					
		*cylB*					
		*cylI*					
		*cylL-l*				+	
		*cylL-s*				+	
		*cylM*				+	
		*cylR1*					
		*cylR2*				+	

The Virulence Factors of Pathogenic Bacteria Database (VFDB) was used to confirm the presence of virulence factors through a BLAST search.

**Table 4:** Multilocus sequence typing of *Enterococcus faecalis* CAUM157.

Locus	Identity	Coverage	Allele
*aroE*	100	100	*aroE*_1
*gdh*	100	100	*gdh*_1
*gki*	100	100	*gki*_1
*gyd*	100	100	*gyd*_7
*pstS*	100	100	*pstS*_9
*xpt*	100	100	*xpt*_1
*yqiL*	100	100	*yqiL*_1

**Figure 2:**
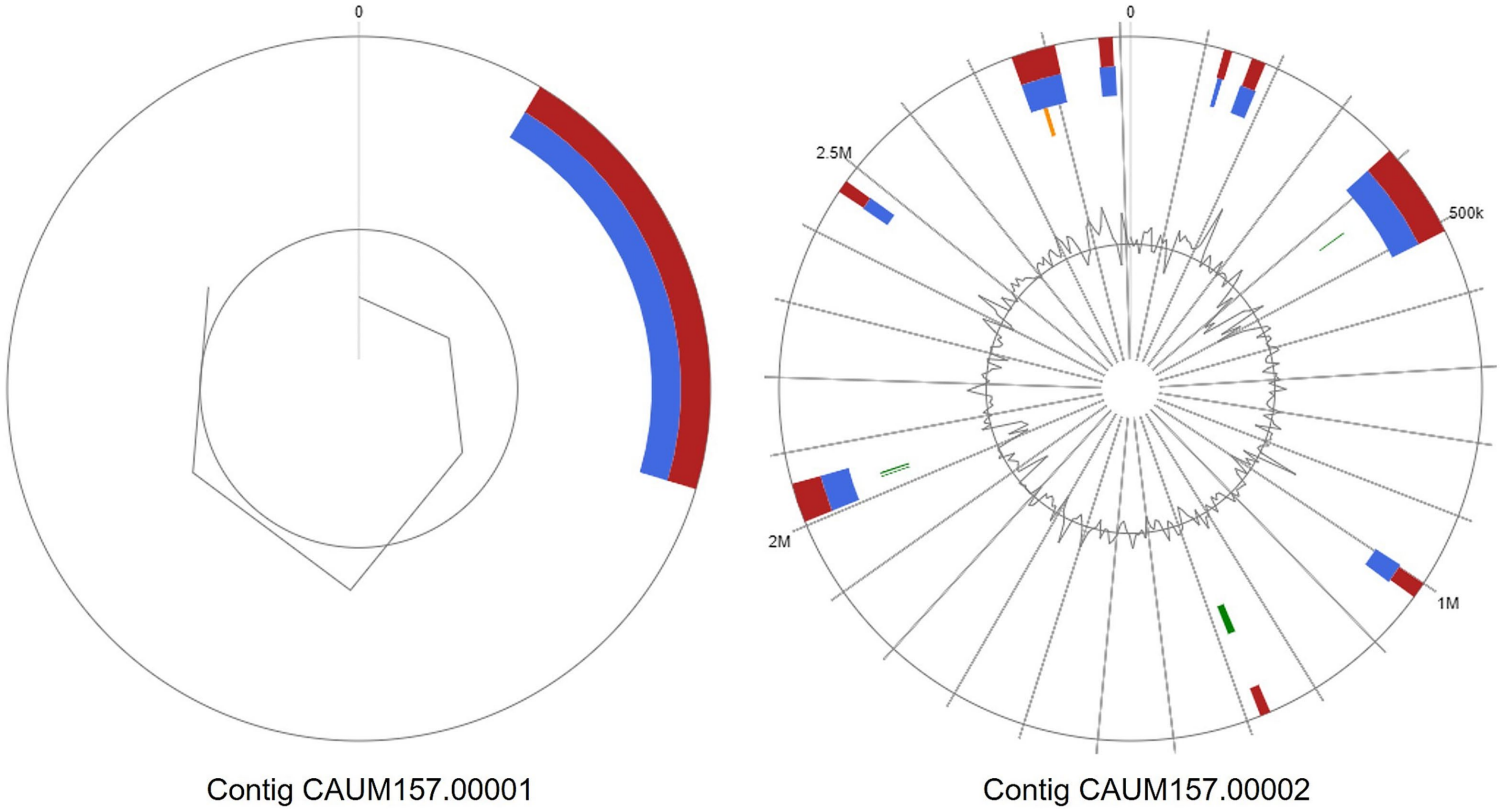
Genomic islands predicted from whole genome sequence of *Enterococcus faecalis* CAUM157 via integrated (red), IslandPath-DIMOB (blue), SIGI-HMM (orange), or IslandPick (green) method.

Moreover, the genome of CAUM157 contains 10 genomic islands (GIs) based on the IslandPath prediction method, as shown in Figure 2. The first contig contained nine GIs with protein coding genes of known and hypothetical functions (Supplementary Data). The GI located at 2,460,659–2,477,436 bp contains the *tet(M)* gene, while the GI located at 2,753,348–2,812,624 bp contains *tet(L)*, *erm(B)*, *aph(3′)-III*, and *ant(6)-Ia* genes, which are associated with antimicrobial resistance. The GI located at 5,104–17,528 bp of the second contig revealed genes encoding the core peptides of enterocin L50, showing high sequence similarity with CAUM157 bacteriocins.

Mobile genetic elements integrated into the genome were also idnetified. Two replicons corresponding to the rep9c (Accession No.: AY855841) and repUS43 (Accession No.: CP0003584) plasmids were detected. The features originally present in the rep9c and repUS43 plasmids were searched against the CAUM157 genome using the BLAST program in EzBioCloud. Plasmid rep9c had 21 of 57 features integrated into the chromosome of CAUM157, whereas plasmid repUS43 had 24 of 44 features (Supplementary Data).

### 3.3 Antimicrobial susceptibility

The antimicrobial susceptibility profile of *E. faecalis* CAUM157 was determined using the broth microdilution method (Table 1). Multidrug resistance, that is, resistance to two or more antimicrobials, was observed in strain CAUM157. Intrinsic resistance to clindamycin, kanamycin, and streptomycin was confirmed along with resistance to ciprofloxacin, erythromycin, and tetracycline. However, the strain was susceptible to ampicillin and vancomycin with MICs of 1 μg/mL and 4 μg/mL, respectively. The observed antimicrobial resistance phenotype was consistent with the antimicrobial resistance genes detected in the genome.

### 3.4 Carbohydrate fermentation profile

The carbohydrate fermentation assay for *E. faecalis* CAUM157 produced a purple-to-yellow color change, indicating a positive result. These results were consistent with those of other *E. faecalis* and *E. faecium* strains (Table 5), as it fermented 22 of 49 test carbohydrates. Based on this analysis, *E. faecalis* CAUM157 does not have a unique fermentable carbohydrate.

**Table 5:** API 50 CH fermentation profile of *Enterococcus faecalis* and *E. faecium* strains.

Carbohydrate	*E. faecalis* CAUM157	*E. faecalis* isolates (El-Gendy et al., 2021)	*E. faecalis* 158B (Solheim et al., 2009)	*E. faecalis* V583 (Solheim et al., 2009)	*E. faecalis* MG5206 (Kim et al., 2022)	*E. faecium* MG5232 (Kim et al., 2022)	*E. faecium* L3 (Kos et al., 2007)
GLY	+	+	+	+	+	+	
LARA					+	+	+
RIB	+	+	+	+	+	+	+
DXYL					+	+	
GAL	+	+	+	+	+	+	+
GLU	+	+	+	+	+	+	+
FRU	+	+	+	+	+	+	+
MNE	+	+	+	+	+	+	+
RHA					+		
MAN	+	+	+	+	+	+	+
SOR	+	+	+	+	+	+	
MDM					+	+	
NAG	+	+	+	+	+	+	±
AMY	+	+	+	+	+	+	±
ARB	+	+	+	+	+	+	±
ESC	+	+	+		+	+	+
SAL	+	+	+	+	+	+	±
CEL	+	+	+	+	+	+	+
MAL	+	+	+	+	+	+	+
LAC	+	+	+	+	+	+	+
MEL							±
SAC	+	+	+	+	+	+	+
TRE	+	+	+	+	+	+	+
MLZ	+	+	+	+	+		
AMD			+		+		
GEN	+		+	+	+	+	±
TUR							+
TAG	+	+	+	+	+		+
GNT	+		+	+	+		

GLY, glycogen; LARA, L-arabinose; RIB, Ribose; DXYL, D-xylose; GAL, galactose; GLU, D-glucose; FRU, D-fructose; MNE, D-mannose; RHA, Rhamnose; MAN, mannitol; SOR, sorbitol; MDM, α-methyl-D-mannoside; NAG, N-acetyl glucosamine; AMY, amygdalin; ARB, arbutin; ESC, esculin; SAL, salicin; CEL, cellobiose; MAL, maltose; LAC, lactose; MEL, melibiose; SAC, saccharose; TRE, trehalose; MLZ, melezitose; AMD, amidon; GEN, α-gentiobiose; TUR, D-turanose; TAG, D-tagatose; GNT, gluconate.

### 3.5 Acid and bile salt tolerance

**Figure 3:**
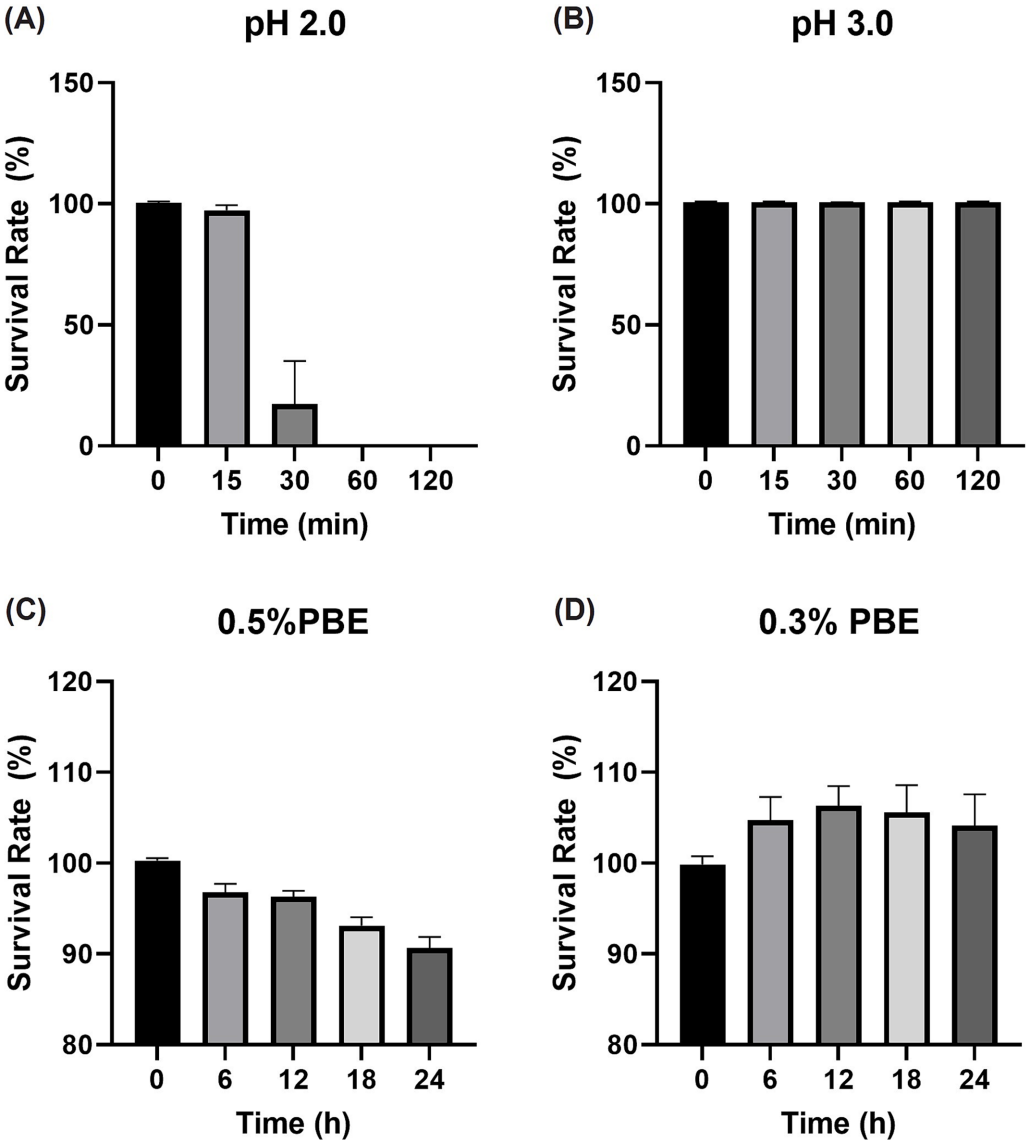
Survival rate of *Enterococcus faecalis* CAUM157 after exposure to artificial gastric juice set to **(A)** pH 2.0 and **(B)** pH 3 and cys-MRS supplemented with **(C)** 0.5% or **(D)** 0.3% porcine bile extract.

Tolerance to acid and bile salts is an important factor that influences the survival, colonization, and functional efficacy of probiotics in the gastrointestinal (GI) tract. Probiotics with acid tolerance can survive passage through the stomach and eventually reach the intestine, where they exert beneficial effects on the host. Figures 3A,B shows that *E. faecalis* CAUM157 could survive exposure to pH 3.0 after 2 h, with a survival rate of 100.72 ± 0.20%. However, after 15 min of exposure to pH 2.0, the survival rate decreased to 97.28 ± 2.19% and continued to drastically drop over time.

In terms of bile salt tolerance, two genes encoding bile salt hydrolase (choloylglycine hydrolase) were identified in the genome of CAUM157 — CM157_00494 (975 bp) and CM157_02577 (1,068 bp). Bile salt activity was assessed using the BSH activity assay. The 5× and 1× cell suspensions of CAUM157 formed white precipitates after 10 and 20 min of incubation in a 37°C water bath, respectively. Additionally, survival of the strain in the presence of 0.3 and 0.5% porcine bile salt revealed the bile salt resistance of the strain, showing survival rates of 104.16 ± 3.42% and 90.65 ± 1.22%, respectively, as shown in Figures 3C,D.

### 3.6 Auto- and co-aggregation with *Listeria monocytogenes*

**Figure 4:**
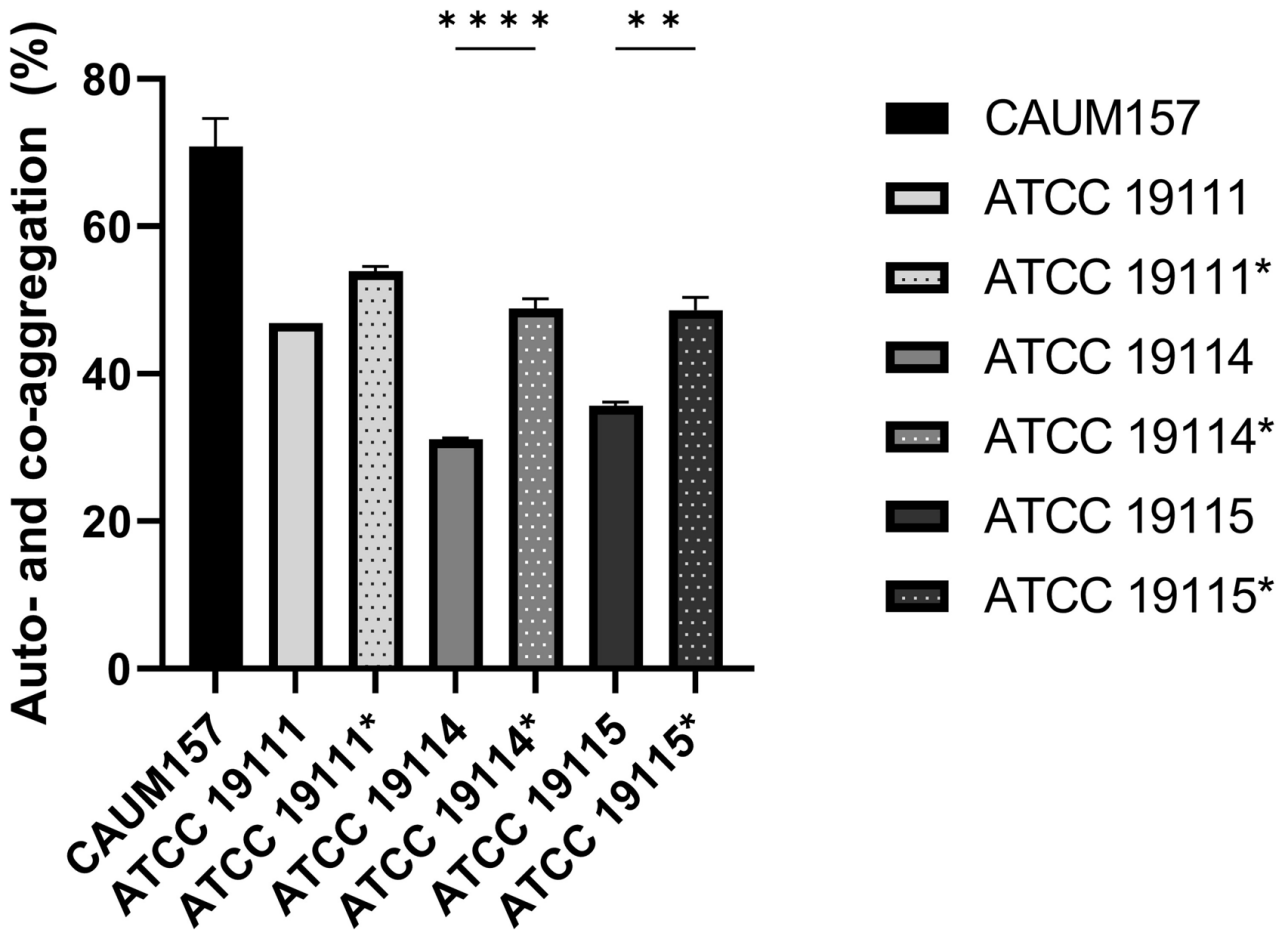
Auto-aggregation of *Enterococcus faecalis* CAUM157 and co-aggregation with *Listeria monocytogenes* ATCC 19111, ATCC 19114, and ATCC 19115. Solid bars represent auto-aggregation and striped bars represent co-aggregation. Statistically significant difference between auto- and co-aggregation is denoted by * based on *α* = 0.05.

The ability of *E. faecalis* CAUM157 to co-aggregate with *L. monocytogenes* was evaluated. Figure 4 shows that CAUM157 has high auto-aggregation ability, with 70.80 ± 6.60% auto-aggregation, whereas *L. monocytogenes* ATCC 19111, ATCC 19114, and ATCC 19115 showed lower auto-aggregation capacity, with 46.92 ± 0.02%, 31.13 ± 0.35%, and 35.60 ± 0.94%, respectively. The combination of CAUM157 with *L. monocytogenes* strains ATCC 19111, ATCC 19114, and ATCC 19115 resulted in a significantly higher degree of aggregation, wherein the degree of co-aggregation measured up to 53.91 ± 1.13%, 48.81 ± 2.31% (*p* < 0.0001), and 48.60 ± 3.05% (*p* < 0.005), respectively. Thus, the auto- and co-aggregation of *E. faecalis* CAUM157 with other bacteria demonstrated bacterial interaction.

### 3.7 Inhibitory activity of M157 bacteriocins

Enterococcal bacteriocins display a broad spectrum of activity and are particularly active against *L. monocytogenes*. The two-peptide bacteriocins produced by CAUM157 (M157A and M157B), previously identified using BAGEL4 (Elnar et al., 2020), effectively inhibited the growth of *L. monocytogenes*, *Clostridium perfringens*, *Streptococcus mutans*, *Staphylococcus aureus*, and to a weaker extent, *Pseudomonas aeruginosa* (Table 6). Based on the MIC, *L. monocytogenes* (MIC, 0.20–0.40 μg/mL) was the most susceptible, followed by *C. perfringens* (MIC, 2.00 μg/mL). The remaining test organisms had MICs ≥ 10 μg/mL.

**Table 6:** Inhibitory activity of CAUM157 bacteriocins against pathogens.

Bacterial Strain	Growth Conditions	Activity*	MIC (μg/mL)
Gram-Positive			
*Listeria monocytogenes* ATCC 19111	TSA, 37°C	++	0.40
*L. monocytogenes* ATCC 19114	TSA, 37°C	++	0.20
*L. monocytogenes* ATCC 19115	TSA, 37°C	++	0.20
*Clostridium perfringens* KCTC 3269^T^	BHI, 37°C	++	2.00
*Cl. perfringens* KCTC 5101	BHI, 37°C	++	2.00
*Cl. perfringens* USDA	BHI, 37°C	++	2.00
*Streptococcus mutans* KCTC 3065^T^	BHI, 37°C	+	10.00
*Strep. mutans* KCTC 5356	BHI, 37°C	+	20.00
*Staphylococcus aureus* ATCC 33591	LB, 37°C	+	10.00
*Staph. aureus* RN 6390	LB, 37°C	+	10.00
Gram-Negative			
*Escherichia coli* ATCC 25922	LB, 37°C	−	−
*E. coli* ATCC 43888	LB, 37°C	−	−
*E. coli* YHS 394	LB, 37°C	−	−
*Pseudomonas aeruginosa* KCTC 1750^T^	LB, 37°C	Weak	>40.00
*P. aeruginosa* KCTC 2657	LB, 37°C	Weak	>40.00
*Salmonella Enteritidis* YHS 383	LB, 37°C	−	−
*Campylobacter jejuni* ATCC 33560^a^	MH, 37°C	−	−
*C. jejuni* ATCC 33291^a^	MH, 37°C	−	−

* Activity was expressed based on the diameter of ZOI: (−) no inhibition; (+) 0.1–5.0 mm; (++) 5.1–10.0 mm; (+++) 10.1–15.0 mm.

a Anaerobic incubation using BD GasPak™ EZ (BD, Australia).

TSA, Tryptic Soy Agar; BHI, Brain-Heart Infusion Agar; LB, Luria-Bertani Agar; MH, Mueller-Hinton Agar.

## 4 Discussion

*E. faecalis* CAUM157 is a bacteriocin-producing strain isolated from raw cow’s milk. The CAUM157 bacteriocin was previously characterized as a Class IIb, two-peptide bacteriocin with high inhibitory activity against *Listeria monocytogenes* (Elnar et al., 2020). The bacteriocin production of *E. faecalis* CAUM157 is considered a favorable trait, particularly for the control of various conditions and diseases, including periodontitis (Park et al., 2021). In the present study, the genome of CAUM157 was examined to gain insights into its safety and potential use as a probiotic strain.

Several virulence factors associated with adherence, anti-phagocytosis, biofilm formation, and enzymes (e.g., gelatinase, hyaluronidase, and serine protease) were detected in the genome of CAUM157, whereas genes related to the bacterial toxin cytolysin (*cylA, cylB, cylI, cylL-1, cylL-s, cylM, cylR1,* and *cylR2*) were not detected. The absence of virulence factors is fundamental to probiotic development. However, certain factors associated with enterococcal virulence are considered advantageous in probiotic strains, as certain virulence factors in pathogenic or opportunistic infections are also detected in probiotic strains. Therefore, referring to these components as “virulence factors” when discussing probiotics may be misleading, given their similar features. Considering this, some studies proposed the concept of “niche factors” (Hill, 2012). Most of these traits are associated with niche establishment, necessary for eliciting their activity — as either probiotics or pathogens (Hill, 2012). These include aggregation factors, exopolysaccharide production, and the proteolytic system (Krawczyk et al., 2021).

Certain virulence factors have potential therapeutic applications. *E. faecium* SF68 (NCIMB 10415), an endogenous intestinal commensal isolate, is a well-studied probiotic *Enterococcus* strain commonly used as an alternative to antibiotics for treating diarrhea. Although the mechanisms underlying the observed probiotic effects remain elusive, one study identified the role of the virulence factor arginine deiminase (ADI) as a potential therapeutic agent. ADI, an enzyme that catalyzes the hydrolysis of peptidyl-arginine, exerts anti-inflammatory and immunomodulatory effects in clinical human and animal trials and is possibly correlated with a reduced duration of acute diarrhea (Ghazisaeedi et al., 2022). As we enter the era of tailored probiotics, this viewpoint offers new avenues for investigating the potential of *Enterococcus* as a genus harboring various virulence factors and their potential use as probiotics in specific contexts. Thus, certain features previously identified as harmful may be useful for treating specific conditions in humans and animals. Furthermore, the identification and expression of putative virulence factors *in vitro* do not yield definitive conclusions regarding the pathogenicity of the strain under natural conditions (Maasjost et al., 2019). For instance, despite harboring the *gelE* gene, CAUM157 failed to degrade gelatin in nutrient gelatin medium (data not shown). Gelatinase is a bacterial protease, specifically an extracellular zinc metalloprotease that contributes to biofilm formation and contributes to the evasion of the host immune system, thus facilitating infections (Del Papa et al., 2007).

Genes encoding antimicrobial resistance were also detected and predicted to confer resistance to aminoglycosides, lincosamides, macrolides, streptogramins A and B, and tetracycline. Notably, all antimicrobial resistance genes are located on genomic islands, including genes for intrinsic resistance to kanamycin, streptomycin, and clindamycin. Furthermore, whole genome comparison between strains of *E. faecalis* revealed that virulence factors and ARGs are common across the species. Resistance to these classes of antibiotics was experimentally confirmed using the broth microdilution method, revealing resistant phenotypes to ciprofloxacin, erythromycin, and tetracycline, while confirming intrinsic resistance to clindamycin, kanamycin, and streptomycin. The antimicrobial resistance profile of *E. faecalis* CAUM157 confers a competitive advantage, considering the common use of antibiotics belonging to the same class in the poultry industry (Muhammad et al., 2019). Nevertheless, the prevalence of ARGs may compromise its application, particularly with the risk of HGT (Meressa and Tseha, 2024). In this regard, careful case-by-case examination is necessary to ensure the safety of the *Enterococcus* strain, and its beneficial properties need to outweigh the risks associated with its potentially pathogenic nature (Hanchi et al., 2018).

To further strengthen the claim that some strains of *E. faecalis* can be potentially used as probiotics, MLST analysis, a molecular typing technique, was performed. The results of this analysis can be used to differentiate strains with regard to quality control and safety. CAUM157 is predicted to be ST21, which is usually of community-based human origin and is generally vancomycin-sensitive (Neumann et al., 2019). The absence of vancomycin resistance genes (*vanA*, *vanB*, and *vanC_2_*) correlates with the sequence type of CAUM157 and is favorable for probiotic development. Although the first report of VRE was from a clinical setting in 1986, its emergence in food-producing animals has been widely associated with the use of avoparcin, an analog of vancomycin (Lawpidet et al., 2021). Medical and public health settings often struggle to manage VRE because of its association with multidrug-resistant infections and persistent colonization (Borgen et al., 2000; Seong et al., 2004; Wada et al., 2022). Since then, efforts have been directed toward preventing an increase in the prevalence of VRE in both clinical and animal settings. As such, susceptibility to vancomycin is crucial, as this drug serves as a “drug of last resort,” and resistance would render the strain unsuitable for probiotic development.

*In vitro* evaluation of the probiotic potential of CAUM157 also revealed promising results. Given the definition of probiotics updated by the International Association for Probiotics and Prebiotics (ISAPP) in 2014, probiotics must be administered in adequate amounts to confer beneficial effects on the host (Hill et al., 2014). In this regard, acid and bile tolerance as well as adhesion to the intestinal mucosa are among the major criteria used to select probiotic candidates because they directly affect the viability of the strain in the GI tract (Tuomola et al., 2001).

Strain CAUM157 showed tolerance to acidic pH and remained viable in the presence of bile, as demonstrated using artificial gastric juice and modified MRS supplemented with bile salts (Vecchione et al., 2018). *In vitro* experiments on the viability of CAUM157 highlighted its potential to survive passage through the gastric environment. In terms of acid tolerance, CAUM157 survived exposure to AGJ (pH 3.0) for 2 h; however, after 30 min of exposure to pH 2.0, the strain was no longer viable. In terms of bile salt tolerance, the viability of CAUM157 was significantly decreased after 6 h of exposure to 0.5% PBE but remained high (> 90%) after 24 h of incubation. In contrast, exposure to 0.3% PBE did not decrease strain viability. Two genes, CM157_00494 (975 bp) and CM157_02577 (1,068 bp), encoding choloylglycine hydrolases or BSH, which are responsible for reducing bile acid toxicity and improving survival in the gut, may also be associated with lipid metabolism. Microbial BSH has been documented to improve gut health by deconjugating bile salts and improving colonization of the GI tract (Wu et al., 2022). Another by-product of bile salt deconjugation is a reduction in blood cholesterol levels. Deconjugated bile acid is more hydrophobic and can be excreted via feces. This process requires utilization of cholesterol for bile production to maintain the bile salt homeostasis in the gallbladder (Kim and Lee, 2005). Thus, the use of CAUM157 as a probiotic with cholesterol-lowering effects deserves further investigation.

The auto- and co-aggregation ability of probiotics is important for bacterial interactions and is the site of action for probiotics in the GI tract (Dlamini et al., 2019). The former pertains to the capacity of bacterial cells of a shared species to attach and aggregate, whereas the latter pertains to the interactions between bacterial cells of distinct species. Strain CAUM157 demonstrated high auto-aggregation and co-aggregation with *L. monocytogenes* ATCC 19111, ATCC 19114, and ATCC 19115. From a probiotic perspective, auto-aggregation can enhance the colonization and persistence of the strain while providing protection against displacement because of the harsh conditions in the gut. Additionally, biofilm formation may be enhanced, which can help establish a niche for probiotic strains. In terms of co-aggregation, the interaction between the probiotic strain and pathogenic bacteria may inhibit the ability of the pathogen to adhere to host cells, thereby reducing its capacity to cause infection (Collado et al., 2007). However, interactions between two probiotic bacteria or with other beneficial bacteria can affect the overall structure and composition of microbial communities, which could promote eubiosis. Therefore, the use of probiotics with efficient auto- and co-aggregation capacities can mitigate the pathogenesis of pathogenic bacteria in the GI tract.

Another promising aspect of probiotics is their ability to utilize nutrients and energy for growth and proliferation in the host. CAUM157 exhibited a wide range of fermentation activities, utilizing 22 of the tested carbohydrates. The end products of fermentation have significant advantages, particularly when considering the potential development of a strain as a feed supplement. This application could result in increased energy provision for the host, leading to enhanced feed conversion efficiency (Rinttilä and Apajalahti, 2013). Several studies have suggested that increased concentrations of short-chain fatty acids (SCFAs), a by-product of fermentation, may modulate the immune system and inhibit pathogenic bacteria (Józefiak et al., 2004; Clavijo and Florez, 2018). For example, Nagpal et al. (2018) demonstrated the ability of a human-origin probiotic cocktail to modulate the gut microbiota and increase native SCFA production, while Mishra et al. (2019) reported the production of propionic acid and butyric acid by *E. faecalis* AG5. Furthermore, bacteriocin production by CAUM157 broadens its application to the inhibition of other potentially harmful bacteria (Elnar and Kim, 2024). The broad activity spectrum of the CAUM157 bacteriocin enables it to inhibit several pathogens, including *Clostridium perfringens* (gas gangrene and diarrhea), *Streptococcus mutans* (dental caries), and *Staphylococcus aureus* (food poisoning in dairy products, septic arthritis) (Franz et al., 2007; Ness et al., 2014). Control of these pathogens is crucial, as they are widespread across a plethora of animals, particularly mammalian and avian species, and have been implicated in various infections and diseases.

## 5 Conclusion

The *in silico* and *in vitro* characterization of *E. faecalis* CAUM157 demonstrated its potential for use in probiotic development. The CAUM157 genome encodes several virulence factors associated with adhesion, biofilm formation, anti-phagocytosis, and various enzymes. Certain antimicrobial resistance genes that confer resistance to aminoglycosides, lincosamides, macrolides, streptogramins A and B, and tetracyclines have also been found. Traditionally, these observations would make *E. faecalis* CAUM157 extremely challenging to develop as a probiotic. However, the advancement of a discipline is accompanied by parallel advancement in its theories and concepts. The concept of labeling “virulence factors” as “niche factors” holds significant importance as it acknowledges that the microbial attributes present in both probiotic and pathogenic bacteria operate similarly. Thus, certain virulence factors of *Enterococcus* should not be viewed as harmful, suggesting that *E. faecalis* can be considered a functional probiotic unless found in an environment that facilitates its ability to exploit and cause harm.

In the case of *E. faecalis* CAUM157, the absence of genes related to cytolysin and vancomycin resistance, as well as its probiotic characteristics and production of broad-spectrum bacteriocins, favors its probiotic development. However, *in vivo* studies are required to evaluate its efficacy, safety, and functionality as a probiotic strain. Given the current state of the field of probiotic studies, a meticulous case-by-case approach for enterococcal probiotic candidates, for both human and animal use, remains the most optimal strategy to ensure the safety and functionality of probiotic candidates.

## Data Availability

The whole genome of *Enterococcus faecalis* CAUM157, a strain isolated from raw cow’s milk, was sequenced, characterized, and deposited into NCBI database with the accession number GCA_014805465.1.
